# Optically Driven Formation of Tailored Phonon Cavities

**DOI:** 10.1002/advs.202514963

**Published:** 2025-10-31

**Authors:** Jianyu Wu, Gaolong Cao, Yuzhu Fan, Saroj P. Dash, Dongkun Yu, Jonas Weissenrieder

**Affiliations:** ^1^ Light and Matter Physics Applied Physics School of Engineering Sciences KTH Royal Institute of Technology Stockholm SE‐100 44 Sweden; ^2^ Department of Microtechnology and Nanoscience Chalmers University of Technology Göteborg SE‐41296 Sweden

**Keywords:** light–matter interaction, phonon cavity, strain, structural dynamics, ultrafast electron microscopy

## Abstract

Optical control of lattice dynamics with high spatiotemporal precision offers a route to manipulate local quantum states—such as magnetic, spin, and topological states—by exploiting the coupling between the lattice and other degrees of freedom. Here, deterministic strain engineering is demonstrated with spatial and temporal characteristics in van der Waals materials using spatially structured femtosecond optical fields. By confining structural oscillations at a submicron scale, phonon cavities with programmable dimensions, oscillation periods, and symmetries are engineered. Through ultrafast electron microscopy analysis and finite‐element simulations the dominant cavity modes, out‐of‐plane confined oscillations, and in‐plane Lamb waves are directly imaged and identified. It is shown that the properties of these phonon cavities are programmable via the spatial profile of the optical excitation, enabling localized modulation of strain and lattice displacement at nanometer and picosecond scales. This work establishes a general framework for spatiotemporal phonon engineering, bridging structured light excitation with atomic‐scale control of lattice dynamics.

## Graphical abstract

It is demonstrated that a structured optical field creates nanoscale “phonon cavities” in van der Waals materials, where atomic vibrations are confined and controlled in space and time. This approach enables programmable control of cavity geometry, symmetry, and frequency, and provides a platform for ultrafast studies of nonequilibrium interactions and transient functionality.

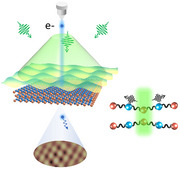

## 1 Introduction

Phonons, the quantized modes of lattice vibrations, are fundamental to the quantum behavior of solids through their coupling to a broad spectrum of collective excitations, such as electronic, spin, and orbital degrees of freedom.^[^ ^1^, ^2^, ^3^ ^]^ This coupling lies at the heart of phenomena such as phonon‐mediated topological phase transitions and phonon‐induced magnetic ordering,^[^ ^4^, ^5^ ^]^ where lattice dynamics can transiently steer the system between distinct quantum states. Unlike equilibrium‐based methods such as chemical doping or strain engineering, the excitation of phonons via optical means enables reversible, non‐equilibrium control of material properties on femtosecond to picosecond timescales.^[^ ^6^, ^7^ ^]^ Leveraging ultrafast temporal control at nanometer spatial resolution offers a powerful platform for manipulating and interrogating complex quantum phases with unprecedented spatiotemporal precision.

Realizing spatiotemporal control requires tools that operate at the intersection of femtosecond dynamics and nanoscale localization. Structured light—optical fields engineered with spatially varying intensity and phase—offers a powerful route to achieve such control. Deliberate interference of laser beams can generate transient optical gratings (TOGs) or lattices (TOLs), producing periodic patterns with tunable symmetry, adjustable periodicity, and nm‐scale control over localized absorbed fluence. Such optical patterns confine light–matter interactions with high spatial and temporal precision and can act as templates for selective excitation of phonons, controlling strain waves, and shaping the spatial potential energy landscape of crystalline solids in ways unattainable through homogeneous excitation.^[^ ^8^, ^9^, ^10^, ^11^, ^12^ ^]^ However, several fundamental questions remain before the approach can be fully leveraged. How do structured optical fields couple to anisotropic materials whose elastic and vibrational responses vary with crystal direction? How do phonons evolve when launched from spatially customized excitation patterns, and how do interference effects shape their propagation and localization? Critically, the inherently spatial nature of such structured excitations demands experimental methods capable of directly visualizing and quantifying the resulting spatiotemporal lattice dynamics with nanometer and picosecond resolution.

In this work, we address these questions by combining ultrafast electron microscopy (UEM) with structured optical fields to design and probe structural dynamics at combined nanometer spatial and picosecond temporal resolution in real space. By interfering multiple femtosecond laser beams, we generate 1D and 2D optical excitation patterns across the surface of van der Waals bonded layered materials. UEM bright and dark field imaging shows that the spatial profile of the optical field dictates the formation of discrete structural oscillations within submicron regions‐formation of phonon cavities. The phonon cavities can be tailored in spatial dimension, symmetry, and frequency of the structural distortion. Through a combination of real‐space imaging and finite element simulations, we identify the phonon modes in cavities, including out‐of‐plane cavity modes and in‐plane Lamb modes. The spatially selective phonon excitation generates coherent structural oscillations and associated confined strain fields within the optically defined boundaries of the cavities. These results demonstrate that spatially structured optical fields can be harnessed to design programmable phonon cavities, facilitating precise modulation of lattice displacements and strain. This approach expands our ability to control dynamic material responses and lays the foundation for optically reconfigurable, transient functional nanoarchitectures.

## 2 Results and Discussion

**Figure 1:**
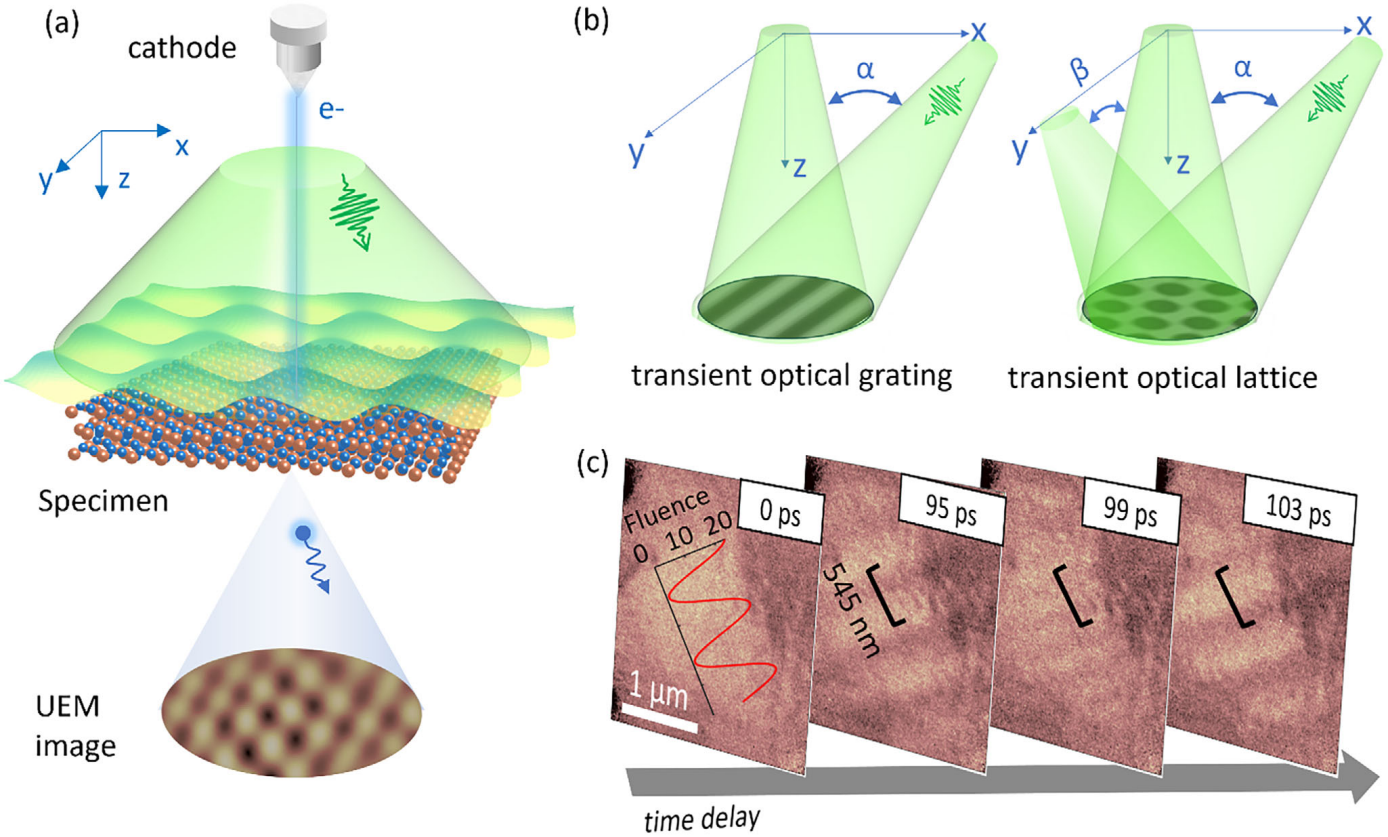
Excitation by transient spatially structured light. a) Schematic illustration of transient optical field excitation of a specimen combined with UEM imaging. The sample is excited by interfering laser beams, and the resulting structural dynamics are probed by electron bunches at variable time delays. b) Schematic illustration of TOG and TOL formation from the interference of two/three incident laser beams. c) UEM dark field snapshots from diffraction spot (210) of PdSe_2_ at selected time delays before and after TOG excitation. First frame merges the calculated laser fluence plot (unit: mJ cm^−2^). Black scale bars indicate the periodicity of the laser‐induced contrast.

**Figure** **1**a illustrates the experimental geometry for nano‐excitation using structured light. A pump laser with a spatially tailored fluence distribution in the *x*‐*y* plane (sample plane) excites the sample. Spatial modulation of the local laser fluence is achieved by splitting the pump laser beam into multiple beams (x, y, and z) and controlling their relative incident angles, as shown in Figure 1b. The interference of the beams at the sample plane generates TOG or TOL. The local fluence follows the relation: $F=I_{1}+I_{2}+2\sqrt{I_{1}I_{2}}\;\cos\left(\varphi_{1}-\varphi_{2}\right)$, where *I_1_* and *I_2_* are the fluence of the two interfering beams and (*φ_1_– φ_2_*) is the phase shift between the beams. Calculations of the local fluence and periodicity for both TOG and TOL are provided in Figure S1, Supporting Information.

The incident laser beam excites the electronic system, initiating rapid energy transfer to the lattice via electron–photon coupling, resulting in lattice distortions.^[^ ^13^ ^]^ Accordingly, the spatial laser fluence distribution directly determines the spatial pattern of the induced lattice distortions. To demonstrate this concept, we investigate two van der Waals materials, a semiconductor PdSe_2_^[^ ^14^, ^15^ ^]^ and an antiferromagnet CrSBr,^[^ ^16^ ^]^ under spatially structured optical excitation. UEM is employed to capture picosecond‐resolved images of the sample following excitation. The electron bunches used for imaging are generated at the UEM cathode by femtosecond laser pulses synchronized with the optical pump. Electrons transmitted or diffracted from the specimen are subsequently recorded to produce bright‐field and dark‐field images. Additional experimental details are provided in the Methods section.

Under excitation of a TOG with a 545 nm periodicity (*λ* = 515 nm, *α* = 70°), the PdSe_2_ sample exhibits periodic lattice distortions, shown by the UEM dark field images from the (210) diffraction spot (Figure 1c; Movie S1, Supporting Information). Figure 1c presents selected frames from a UEM dark‐field imaging time sequence. The image collected at 0 ps shows the flat morphology of the sample. At a positive time delay, periodic contrast fringes emerge, whose intensity and spacing oscillate over time. The observed periodicity and orientation of the structural distortions match the interference geometry of the exciting TOG. These structural modulations are driven by optical excitation of coherent phonon modes. The structured‐light excitation confines the generation of phonons to one dimension, i.e., the phonon density is periodically modulated along the interference direction while remaining constant along the interference fringes. In contrast to homogeneous laser excitation, where phonons are excited uniformly or generated at localized hot spots such as defects or sample edges,^[^ ^17^, ^18^ ^]^ structured light illumination produces a spatially periodic excitation, launching coherent plane wave phonon fronts. This excitation geometry eliminates the requirement of defects to localize the generation of phonons and enables controlled studies of phonon propagation in otherwise uniform samples. The resulting local lattice modulations arise from the propagation and mutual interference of the modes. This observation confirms the imprinting of lattice distortions via optical excitation.

**Figure 2:**
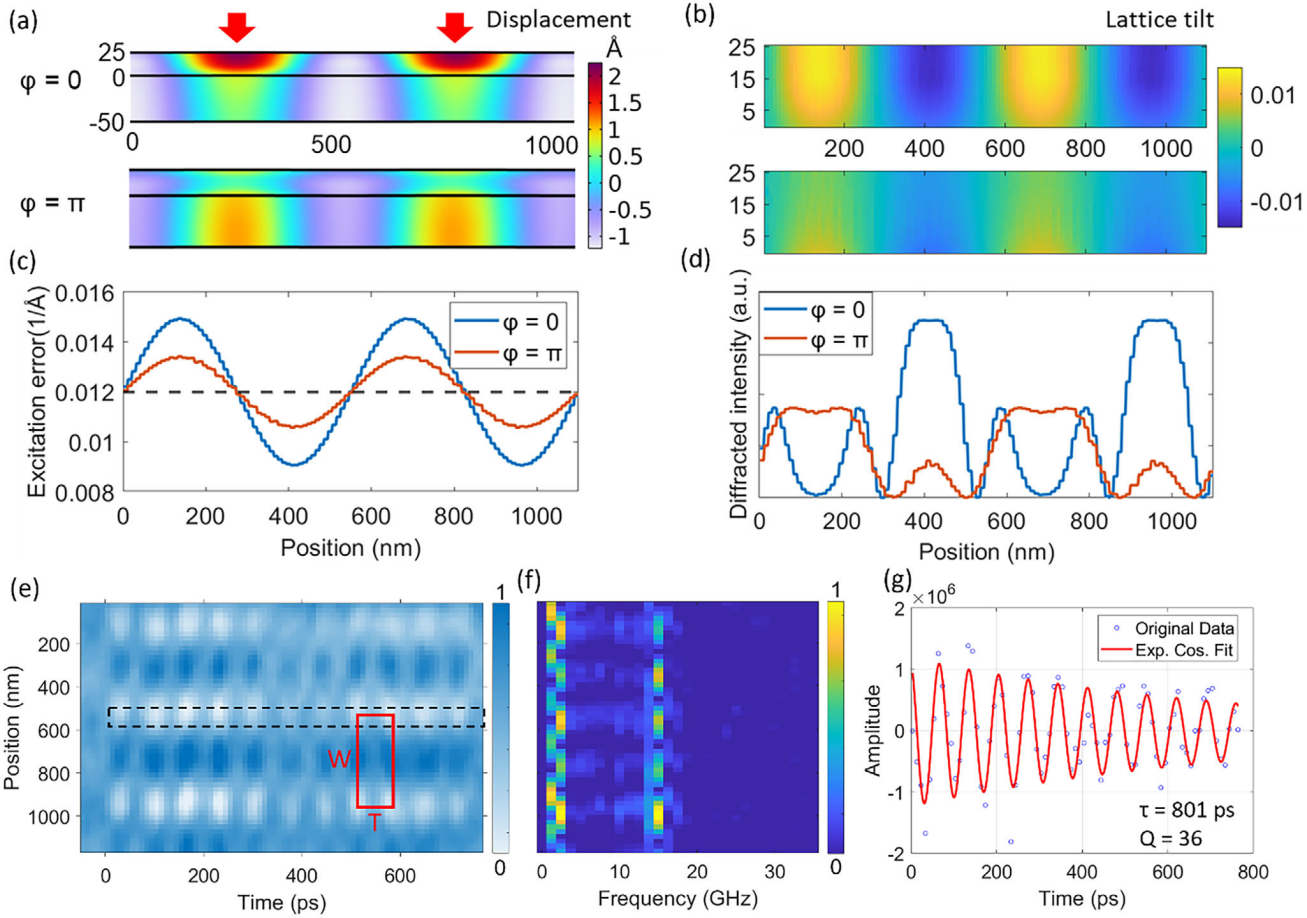
Optically induced structural distortions. a) Finite element simulations of out‐of‐plane displacements (in Å) of a 25 nm PdSe_2_ on a 50 nm Si_3_N_4_ substrate (cross section) at different phase φ of the oscillation. Red arrows indicate positions of constructive interference in the TOG. b) Local lattice tilt of PdSe_2_ from the gradient of displacement in (a). c) Spatial dependent excitation error from the local lattice tilt. The excitation error is averaged over the thickness direction of the sample. The gray dashed line indicates the excitation error in a static state. d) Simulated spatial diffracted intensity of Bragg spot (210), with spatial coordinate perpendicular to the TOG fringes. e) STCP constructed from a time‐resolved UEM image series of CrSBr under TOG excitation. The red rectangle indicates a phonon cavity with spatial dimensions W and temporal dimension T. f) 2D frequency map constructed by applying FFT on the pixel intensity for each row in STCP in (e). g) Extracted intensity profile from the dashed rectangle in panel (e). Red solid line is a fit to an exponential decay cosine function.

In order to determine how the lattice dynamics govern the observed UEM image contrast, we compared the experimental results to finite element modelling (FEM). Details of the simulations are provided (Figure S7, Supporting Information). **Figure** **2**a shows two phases (*φ* = 0 and *π*) of the structural oscillation that follow excitation. At *φ* = 0 we observe strong distortion localized near the top surface, while at *φ* = *π* distortion is most prevalent at the bottom surface of specimen. These out‐of‐plane displacements follow the exciting TOG periodicity, with the largest displacements occurring in the regions of highest local fluence. The amplitude of the out‐of‐plane (*z*‐direction) displacements (≈2 Å) is approximately four times larger than in‐plane (*x*–*y* plane) displacements, consistent with the anisotropic elastic properties of the van der Waals material.^[^ ^19^ ^]^ The laser‐induced local lattice distortions result in a spatial modulation of the local lattice tilt. The local lattice tilts will, in turn, result in a spatial modulation of the excitation error (*s*). The intensity at the Bragg spot will depend on the local excitation error according to:^[^ ^20^ ^]^

(1) $$I\left(s\right)=\frac{1}{1+{\left(s\xi_{g}\right)}^{2}}sin^{2}\left(\pi t\sqrt{s^{2}+\frac{1}{{\xi_{g}}^{2}}}\right)$$

where *ξ_g_* is the extinction distance and t is the specimen thickness. Direct measurements of the extinction distance for PdSe_2_ are not available; we therefore estimate the value of ξ_g_ ≈ 40 nm from Cu and Au (200) Bragg reflections.^[^ ^21^ ^]^ The simulated local tilts, extracted from a spatial distribution of the displacement gradient (Figure 2b), show that the highest tilt occurs in‐between regions of high and low excitation fluence. The local tilt angles will depend on the phase of the oscillation, as shown in Figure 2c, leading to local intensity oscillations in the dark field images (Figure 2d). The simulated images reproduce the UEM observation (Figure S13, Supporting Information).

The space time contour plot (STCP) in Figure 2e shows the spatiotemporal evolution of structural distortions in CrSBr following TOG excitation. Repeating units in both space and time dimensions can be observed, indicated by the red rectangle. The spatial dimension of the unit (*W*) is identical to the spatial periodicity of TOG, and its temporal period (*T*) is ≈70 ps. A frequency map from a fast Fourier transform (FFT) of the time series, Figure 2f, shows that the structural oscillations (high FFT amplitude) are localized at the center and edges of each unit, leaving other regions relatively static. Two dominant modes are identified at ≈2 and 14.4 GHz, corresponding to temporal periods of ≈500 and ≈70 ps, respectively. As discussed in the following section, these are assigned to Lamb and cavity modes. Because the excitation of phonons is confined to local regions, we label this optically engineered, spatially confined vibrational domain as a virtual phonon cavity.^[^ ^22^, ^23^, ^24^, ^25^ ^]^ The oscillation amplitude of this phonon cavity is extracted from the STCP (Figure 2g). The results can be fitted by an exponential decay cosine function: *I*(*t*) = *Ae* ^−*t*/τ^cos (2π*ft* +  φ) + *y*_0_, where *A* is the oscillation amplitude, *t* is the time delay, *τ* is the exponential decay time constant, *f* is the oscillation frequency, *φ* is the phase, and *y_0_* is an offset. The fitted decay constant *τ* is 801 ps. The quality factor of the cavity mode at *f* = 14.4 GHz can be calculated from *Q* =  πτ*f* = 36.^[^ ^26^ ^]^

What separates the cavities demonstrated here from conventional physically fabricated cavities is that the dimensions of the cavities are defined by the spatial profile of the local laser fluence. The spatial dimension *W* is controlled by the TOG periodicity and can be tuned precisely by adjusting the pump wavelength or the angle between the interfering beams. The temporal dimension *T* dependents on parameters such as film thickness, intrinsic material properties, and excitation fluence for high harmonic phonon modes excitation. This flexibility enables the creation of programmable structural nano‐oscillators, where cavity size and frequency can be tailored on demand. Examples of engineered phonon cavities with *W* between 545 and 1000 nm and 𝑇 spanning 25–71 ps are shown in Figure S4 (Supporting Information).

**Figure 3:**
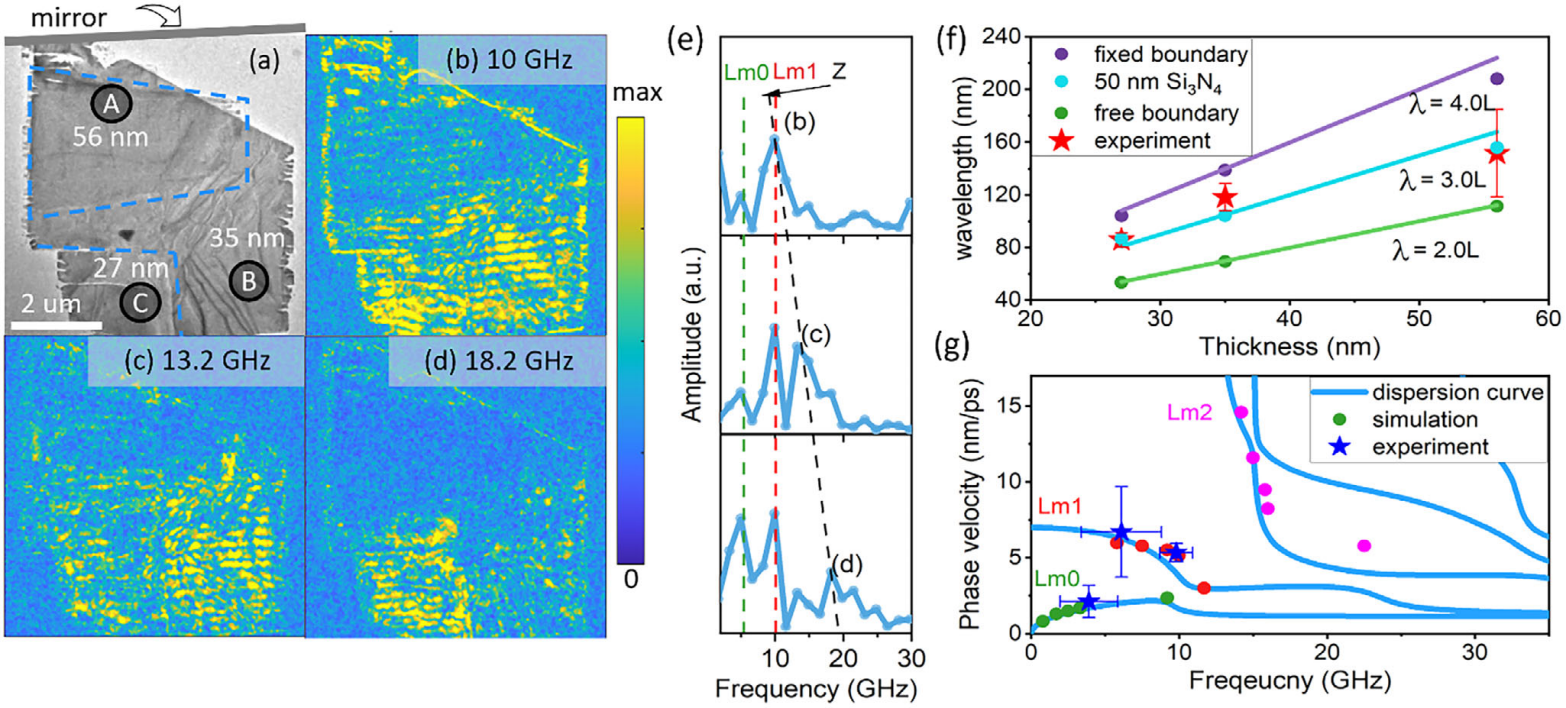
Survey of excited phonon modes. a) Bright‐field image of a PdSe_2_ sample with three zones of different thickness. The dashed lines indicate the boundaries between zones of different thickness. The zones are labeled A (thickness: 56 nm), B (35 nm), and C (27 nm). b–d) FFT amplitude maps from a time series of UEM images. The FFT is applied to each pixel of the image series. Amplitudes at frequencies 10.0 (b), 13.2 (c), and 18.2 GHz (d) are displayed on a false color scale. e) Frequency spectra obtained by performing FFT of the intensity traces extracted from zones A, B, and C in (a). The dashed lines in different colors divide peaks into different branches of phonon modes. The peaks used for FFT mapping are labeled. f) Wavelength dependence of z phonon modes on sample thickness and boundary conditions from FEM simulations (filled circles) and experiments (red stars). Wavelength is calculated by *λ* = v/f, where v is the wave velocity (1.56 nm ps^−1^) and f is the frequency from the FFT spectra. Ideal wavelength‐thickness dependence is plotted as a guide to the eye (solid lines). g) Lamb wave dispersion diagram and data points from FEM simulations (filled circles) and experiment (stars). Error bars are estimated from the full width at half maximum of the FFT peaks.

To obtain further understanding of the oscillatory behavior of the phonon cavities, we investigated phonon modes in regions of different local thicknesses by comparing experiments and simulations. A PdSe_2_ flake containing three domains (A–C) with different thicknesses was selected for TOG excitation. A bright‐field image of the sample is shown in **Figure** **3**a. The thicknesses of the three regions (56, 35, and 27 nm) were determined using electron energy loss spectroscopy (EELS). The TOG was projected onto the sample with a fringe direction aligned with the mirror configuration indicated in Figure 3a. The experimental geometry is described in greater detail in Figure S2 (Supporting Information).

FFT analysis of intensity fluctuations in the UEM image sequence (Movie S2, Supporting Information) reveals three distinct phonon branches (Figure 3e). The branch indicated by a black dashed line exhibits a sample thickness dependence. This trend is further visualized in the FFT maps of Figure 3b–d: domain A oscillates at 10.0 GHz; domain B at 13.2 GHz, and domain C at 18.2 GHz. The oscillation period exhibits a linear relationship with sample thickness (Figure 3f), which is indicative of a cavity mode (z mode) in the thickness direction. For this mode, the wave fronts propagate along the film thickness direction and interfere with reflections from the top and bottom surfaces to form standing waves. The boundary conditions restrict the allowed wavelengths to λ = 2*L*/*n* for a free standing film and to λ = 4*L*/*n* when one boundary is fixed, where *L* is the film thickness and *n* is an integer. Our FEM simulations reproduce the thickness‐dependent oscillations under different boundary conditions (Figure 3f). For the PdSe_2_ flake supported on a 50 nm Si_3_N_4_ substrate, the coupling to the substrate modifies the standing wave condition, shifting the fundamental z mode (*n* = 1) wavelength to ≈λ = 3*L*. Additional simulations show how the wavelength of the z mode increases continuously with increasing substate thickness (Figure S8, Supporting Information). The z mode induces structural distortion in both the in‐plane and out‐of‐plane directions. The in‐plane displacement is symmetrically distributed around the cavity center (Figure S9, Supporting Information). In contrast, the out‐of‐plane displacements diminish gradually from the cavity center to the edges. Both the in‐plane and out‐of‐plane displacements exhibit the same periodicity as the cavity. Higher order harmonic cavity modes (second and third order) are also observed (Figure S4, Supporting Information), highlighting the potential of using optically driven phonon cavities as a playground of nonlinear phenomena, such as tunable intermodal coupling.

In addition to the z mode, two thickness‐independent phonon branches at 4 GHz (Lm0) and 10 GHz (Lm1) were observed. These modes depend strongly on the TOG periodicity (Figure S3, Supporting Information) and correspond to in‐plane guided modes. The extracted phonon velocities match the dispersion diagram of Lamb wave modes Lm0 and Lm1, as shown in Figure 3g. Lamb waves are guided plate modes with displacement components in both in‐plane and out‐of‐plane directions (Figure S12, Supporting Information). Because the PdSe_2_/Si_3_N_4_ composite structure breaks out‐of‐plane symmetry in the thickness direction, the excited Lamb modes do not have a clear symmetric or antisymmetric character.^[^ ^27^ ^]^ We therefore denoted them as “Lm” with different orders. FEM simulations accurately reproduce the dispersion diagram for the first three Lamb branches (Figure S10, Supporting Information). TOG excitation coherently drives Lamb waves perpendicular to the fringes. The propagation of these waves produces constructive interference both at the TOG fringes and at the midpoints between fringes, resulting in maximum out‐of‐plane displacement at the cavity center and edges. The total structural displacement within the phonon cavities represents the combined contributions of Lamb and cavity modes. Although the coupling between Lamb modes and cavity modes is not observed in the present results, such coupling could, in principle, be achieved by tuning the excited phonon frequencies or via parametric excitation.

**Figure 4:**
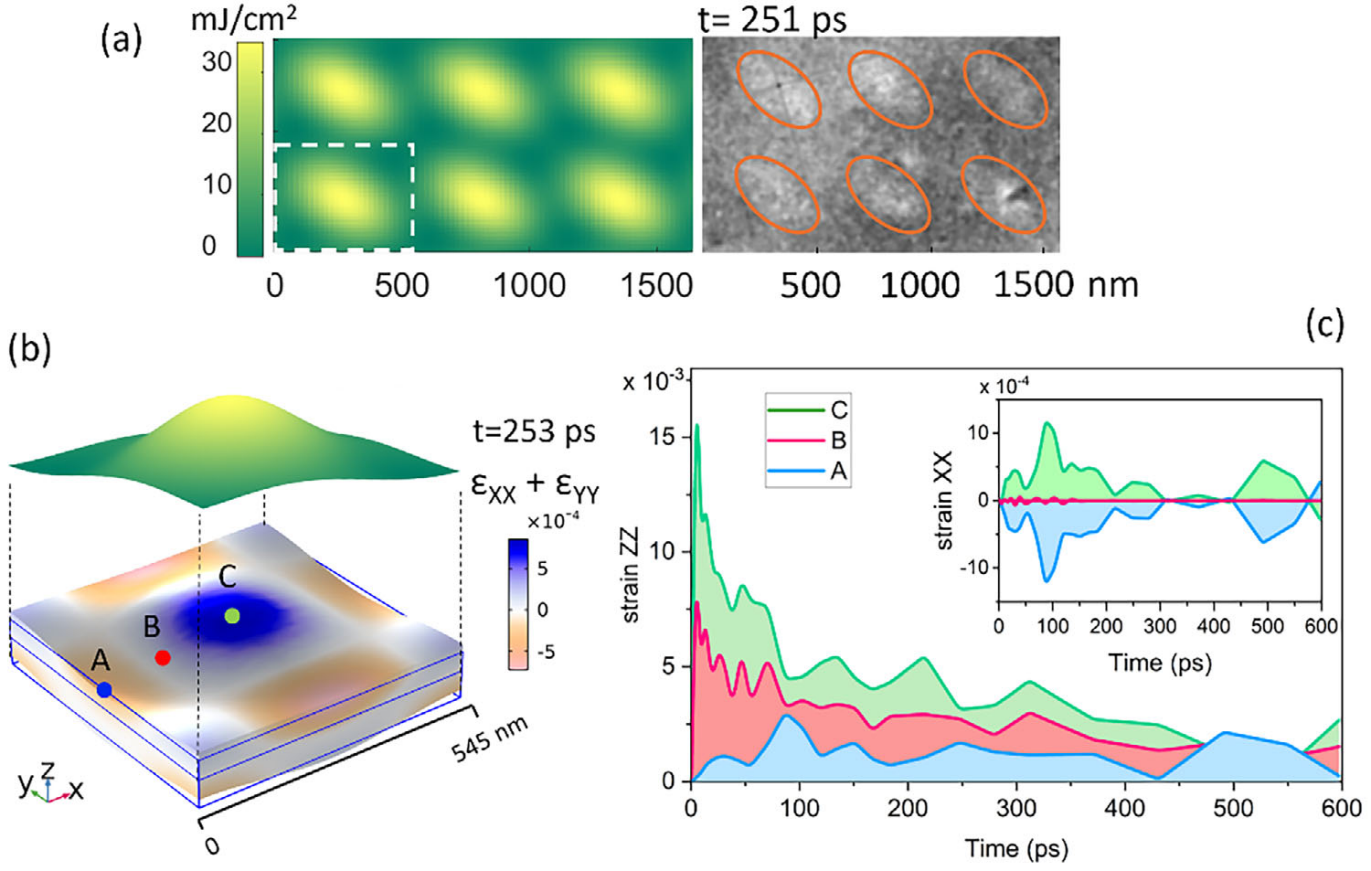
Strain field modulation by TOL excitation. a) Left: The laser fluence distribution in the TOL for a 1000 × 1600 nm region. Right: UEM snapshot (at 251 ps delay) from a PdSe_2_ sample excited with a TOL. Orange ellipses serve as a guide to the eye for indicating local fluence maxima. b) In‐plane strain profile (ε_xx_ + ε_yy_) and incident laser fluence distribution in one excitation unit. Three points in blue (A), red (B), and green (C) were selected for tracing strain‐time profiles. c) Strain‐time profiles for the stain tensor elements ε_zz_ and ε_xx_ at points A–C.

The dimensionality of a phonon cavity can be controlled through multiple‐dimensional structured optical fields. The left panel of **Figure** **4**a shows the excitation profile of a TOL with 545 nm periodicity, generated by three 515 nm laser beams intersecting at incident angles *α* = *β* = 70°. This excitation geometry produces a square TOL featuring elliptical local fluence maxima. The corresponding UEM bright field image of a PdSe_2_ sample at 251 ps delay is shown in the right panel of Figure 4a. Because the excitation fluence varies in two dimensions, phonon cavities are formed in the *x*–*y* plane. Selected‐area electron diffraction confirms the resulting 2D structural modulation (Figure S6, Supporting Information). Analogous to 1D cavities, the central and edge regions of the 2D phonon cavities oscillate in opposite phase, while the intermediate regions remain nearly stationary.

The ability to modulate lattice dynamics at the nanoscale enables precise control over local material properties. Strain, in particular, plays a critical role due to its strong coupling with electronic and optical characteristics. FEM simulations show that TOL excitation in PdSe_2_ produces a well‐defined 2D strain cavity, as illustrated in Figure 4b. Within each cavity, the in‐plane strain components (*ε*_xx_ and *ε*_γγ_) exhibit alternating regions of maximum compression and tension near the center and edges, respectively, separated by zones of zero strain. The temporal *ε*_xx_ profiles in the inset of Figure 4c show that these minimal‐strain zones remain nearly stationary throughout the measurement, while the regions within local fluence maxima or minima exhibit pronounced oscillations. For out‐of‐plane strain (*ε*_zz_), the magnitude increases progressively with laser fluence from the cavity boundary toward its center, and decays over time, as shown in Figure 4c. These results demonstrate that structured optical fields can generate nanoscale strain cavities, providing ultrafast spatiotemporal control of material properties.

The phonon cavity defined in this work is an optically engineered, spatially confined vibrational domain in which coherent phonons are localized within constructive interference regions of a TOG or TOL. Unlike conventional standing waves in a thin film, these optically defined cavities have deterministic dimensions both in the thickness direction and in the plane of the sample, providing an efficient method to control the effective mode volume. Crucially, the cavity geometry is reconfigurable: by changing the optical interference pattern, the spatial size, symmetry, and excitation amplitude of the cavity can be rewritten on ultrafast timescales, enabling programmable confinement of lattice oscillations. The optically formed z‐mode cavities reported here (*Q* ≈ 36, *τ* ≈ 800 ps) exhibit modest performance compared with high‐Q phononic resonators fabricated by lithography. Although nanofabricated resonators can achieve higher *Q* values, they are limited by pre‐determined fixed geometries and high fabrication complexity.^[^ ^25^, ^28^ ^]^ The non‐invasive, dynamically reconfigurable cavity control through TOG/TOL excitation enables unique opportunities to study nonequilibrium phonon–quasiparticle interactions,^[^ ^2^, ^5^ ^]^ light‐driven phase transitions, and reconfigurable optomechanical functionality. Furthermore, the excitation of Lamb waves is also modulated by the structured optical field. The superposition of phonons launched from multiple excitation sites leads to interference that concentrates vibrational energy at antinodes. With appropriate excitation geometry, this mechanism can effectively confine in‐plane modes into oscillators supporting predominantly lateral motion. Because of the low frequencies of Lamb‐modes, we could not extract reliable values of *Q* from the current observation time window. Nevertheless, the spatial confinement and mode structure are clearly resolved. Finally, we note several realistic pathways to increase *Q* in future work, including substrate engineering or suspension to reduce acoustic leakage, materials selection, cryogenic operation to suppress anharmonic damping, and laser‐field design for in‐plane mode frequency selection.

## 3 Conclusion

This work demonstrates that structured optical fields can precisely control nanoscale phonon dynamics utilizing ultrafast electron microscopy. By employing TOG/TOL on van der Waals materials, we report four key advances: 1) the formation of phonon cavities characterized by deterministic spatial patterns of structural oscillation and static deformation; 2) the resolution of anisotropic phonon propagation, distinguishing contributions from out‐of‐plane and in‐plane modes to the lattice distortion; 3) the quantification of strain‐field modulation as a function of optical excitation parameters; and 4) the programmability of phonon cavities through tailored optical field profiles and sample‐specific properties. These findings establish a platform for investigating coherent phonon engineering, enabling the design of topological phononic states, strain‐mediated quantum phenomena, and ultrafast optomechanical devices. By bridging structured light excitation with atomic‐scale structural control, our approach offers a general framework for tuning material properties through light‐induced lattice dynamics in quantum materials and van der Waals heterostructures.

## 4 Experimental Section

Single crystal samples were purchased from 2D Semiconductors, USA (PdSe_2_) and HQ Graphene, Netherlands (CrSBr). The thin film sample was prepared using mechanical exfoliation of bulk material and dry transfer onto a 50 nm Si_3_N_4_ TEM grid. The sample was illuminated by 300 fs pulsed laser at a wavelength of 515 nm/1030 nm. The exciting laser beam was aligned to be partially reflected by a slanted Si surface of a TEM grid, which was coated with aluminum for improved reflectivity. For TOG generation, one slanted Si surface was employed for partial reflection of the laser beam. For TOL generation, two perpendicular slanted Si surfaces, in close proximity to the sample, were employed for partial reflection of the pump laser beam. The resulting laser fluence distribution was governed by the amplitude and phase difference of the interfering beams. The phase difference was determined by the optical path difference between the direct and reflected beams. Additional details for TOG/TOL generation and local laser fluence calculations are provided in the Supplementary Material. The UEM experiments were performed using a modified JOEL JEM‐2100 TEM.^[^ ^29^ ^]^ The electrons were detected using a hybrid pixel detector (CheeTah T3, Amsterdam Scientific Instruments).

## Conflict of Interest

The authors declare no conflict of interest.

## Supporting information



Supporting Information

Supplemental Movie 1

Supplemental Movie 2

## Data Availability

The data that support the findings of this study are available from the corresponding author upon reasonable request.
