# Supplementary material for: Optically Driven Formation of Tailored Phonon Cavities
Source: Adv Sci (Weinh). 2025 Oct 31;13(4):e14963. doi: 10.1002/advs.202514963 (PMC12822412; doi:10.1002/advs.202514963)
Supplement: Supplementary file 1 — Supporting Information [file ADVS-13-e14963-s003.docx]

Supporting information

**Optically driven formation of tailored phonon cavities**

*Jianyu Wu, Gaolong Cao, Yuzhu Fan, Saroj P. Dash, Dongkun Yu, Jonas Weissenrieder **

**Laser Fluence Calculation for transient optical grating/lattice**

**
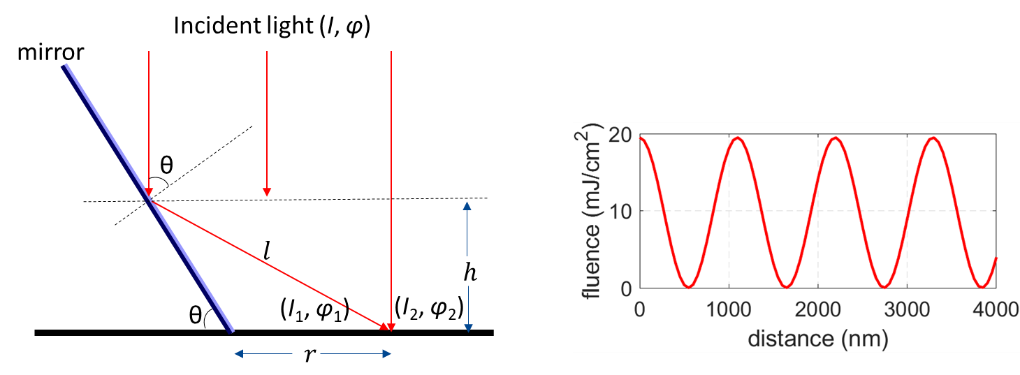
**

Figure S1. left: sketch of two laser beams interference. Right: fluence distribution as a function of distance r from the mirror.

For two incident beams (Transient Optical Grating, TOG), the local laser fluence *F* resulting from the interference at position *r* is given by:

$$\boldsymbol{F=}\boldsymbol{I}_{\boldsymbol{1}}\boldsymbol{+}\boldsymbol{I}_{\boldsymbol{2}}\boldsymbol{+2}\sqrt{\boldsymbol{I}_{\boldsymbol{1}}\boldsymbol{I}_{\boldsymbol{2}}}\boldsymbol{cos(}\boldsymbol{\varphi}_{\boldsymbol{1-}}\boldsymbol{\varphi}_{\boldsymbol{2}}\boldsymbol{)}$$

Similarly, for three-beam interference (Transient Optical Lattice, TOL), the total fluence *F* is given by:

$$\boldsymbol{F=}\boldsymbol{I}_{\boldsymbol{1}}\boldsymbol{+}\boldsymbol{I}_{\boldsymbol{2}}\boldsymbol{+}\boldsymbol{I}_{\boldsymbol{3}}\boldsymbol{+2}\sqrt{\boldsymbol{I}_{\boldsymbol{1}}\boldsymbol{I}_{\boldsymbol{2}}}\boldsymbol{cos}\left( \boldsymbol{\varphi}_{\boldsymbol{1-}}\boldsymbol{\varphi}_{\boldsymbol{2}} \right)\boldsymbol{+2}\sqrt{\boldsymbol{I}_{\boldsymbol{1}}\boldsymbol{I}_{\boldsymbol{3}}}\boldsymbol{cos}\left( \boldsymbol{\varphi}_{\boldsymbol{1-}}\boldsymbol{\varphi}_{\boldsymbol{3}} \right)\boldsymbol{+2}\sqrt{\boldsymbol{I}_{\boldsymbol{2}}\boldsymbol{I}_{\boldsymbol{3}}}\boldsymbol{cos(}\boldsymbol{\varphi}_{\boldsymbol{2-}}\boldsymbol{\varphi}_{\boldsymbol{3}}\boldsymbol{)}$$

where:

- *I₁*, *I₂*, and *I₃* are the incident fluences of the three beams,
- *φ_1_*, *φ_2_* and *φ_3_* are the phase of the respective beam at position *r*.

The phase difference between a direct beam and a beam reflected by a slanted mirror is given by:

$$\boldsymbol{\varphi}_{\boldsymbol{1-}}\boldsymbol{\varphi}_{\boldsymbol{2}}\boldsymbol{=}\frac{\boldsymbol{2}\boldsymbol{\pi}\left( \boldsymbol{l-h} \right)}{\boldsymbol{\lambda}}\boldsymbol{=}\frac{\boldsymbol{2}\boldsymbol{\pi r}}{\boldsymbol{\lambda}}\frac{\boldsymbol{sin\theta(1+cos}\boldsymbol{2}\boldsymbol{\theta)}}{\boldsymbol{cos\theta}}$$

where:

- *θ* is the angle of the slanted mirror, 55° for this setup,
- *λ* is the wavelength of the pump laser,
- *r* is the distance from the interference point to the edge of the mirror.

For an average incident fluence at 5 mJ/cm^2^, the fluence distribution for the interference of two beams is a cosine function of distance *r* from the mirror, as plotted in right panel of Figure S1. For 3 beams interference, the fluence is in lattice geometry featuring elliptical local fluence maxima, as shown in main text Figure 4.

**Calculation of excitation error**

The excitation error is the difference between the scattered wavevector magnitude and the incident-wave magnitude along the Ewald-sphere direction. The excitation error *s* is calculated according to:

$$s=\left| \vec{k}+\vec{g} \right|-\left| \vec{k} \right|\approx\frac{2\vec{k}\cdot\vec{g}+g^{2}}{2k}$$

Where *k* is the incident electron wavevector and *g* is the reciprocal-lattice vector. For Bragg spot (210), $\vec{k}\cdot\vec{g}=0$, $\left| \vec{g} \right|\approx2.44$ Å^-1^. For electron at 200 keV, $\left| \vec{k} \right|=2\pi/\lambda\approx250.53$ Å^-1^, then *s* = 0.0118 Å^-1^.

**Additional results:**


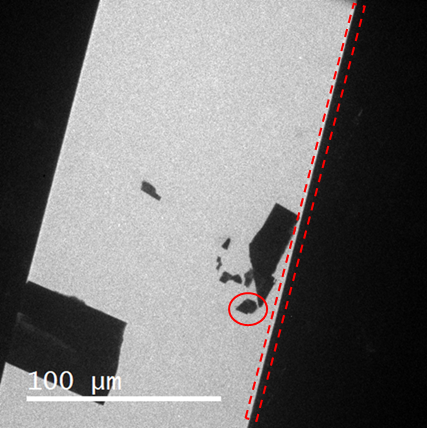


Figure S2. Sample position (red ellipse). The Si surface of the TEM grid (in dashed rectangle) serves as mirror for partial reflection of the incident laser. The incident laser beam (beam diameter 200 μm) is projected to the selected sample. The direct incident and reflected laser beams generate a transient optical grating on the sample through interference.
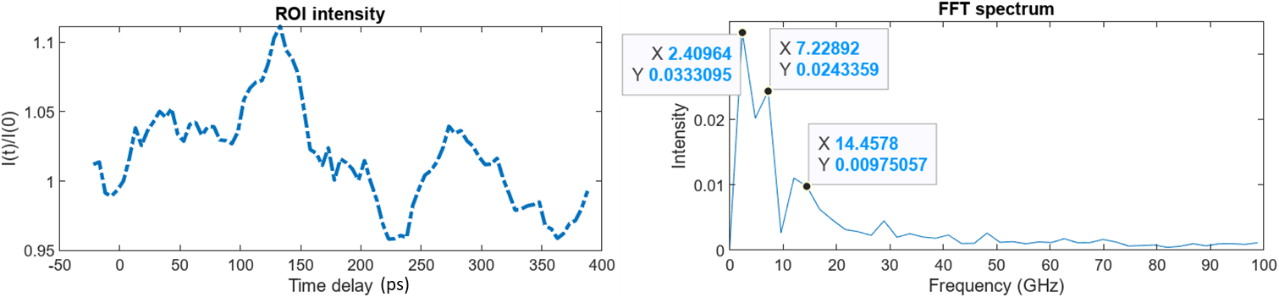
Figure S3. Left: temporal intensity trace for a 35 nm thickness PdSe_2_ sample from dark -field images collected from the (210) Bragg spot. The sample was pumped by a TOG at a wavelength of 1030 nm. The laser fluence before interference is 6.4 mJ/cm^2^. Right: the frequency spectrum from of intensity trace, Lm 1 mode at 7.2 GHz and z mode at 14.5 GHz.


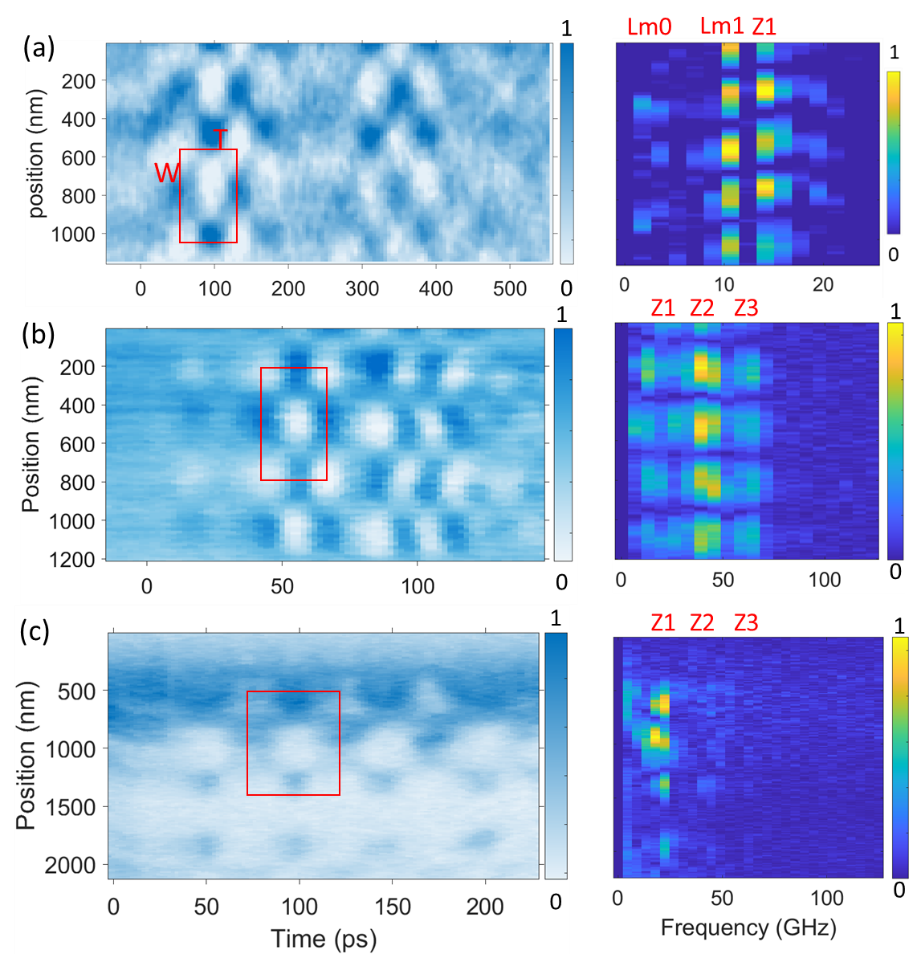


Figure S4 (a)-(c) Space time contour plots (STCP) of structural oscillations in PdSe_2_ samples pumped by TOGs and corresponding frequency maps. Red rectangles highlight the phonon cavities in space-time domain. Phonon modes are highlighted for Lamb mode (Lm0, Lm1, too low to resolve in b and c) and cavity mode in different harmonics (z1, z2, z3).

The spatial size of the phonon cavity is tunable with the periodicity of the optical grating (b, c). The oscillation frequency is tunable with the sample thickness (a, b) and laser fluence (b, c). The harmonic z modes are generated in (b) because of the higher absorption for 515 nm laser.[1] The excitation condition is provided in the following table.

Table S1. Summary of phonon cavities and laser excitation in Figure S4.

| **Frame** | **W (nm)** | **T (ps)** | **Laser wavelength (nm)** | **Laser fluence (mJ/cm2)** | **Sample (thickness, nm)** |
| --- | --- | --- | --- | --- | --- |
| a | 545 | 71 | 515 nm | 7.5 | PdSe_2_ (36) |
| b | 545 | 25 | 515 nm | 4.5 | PdSe_2_ (24) |
| c | 1000 | 50 | 1030 nm | 5.3 | PdSe_2_ (24) |


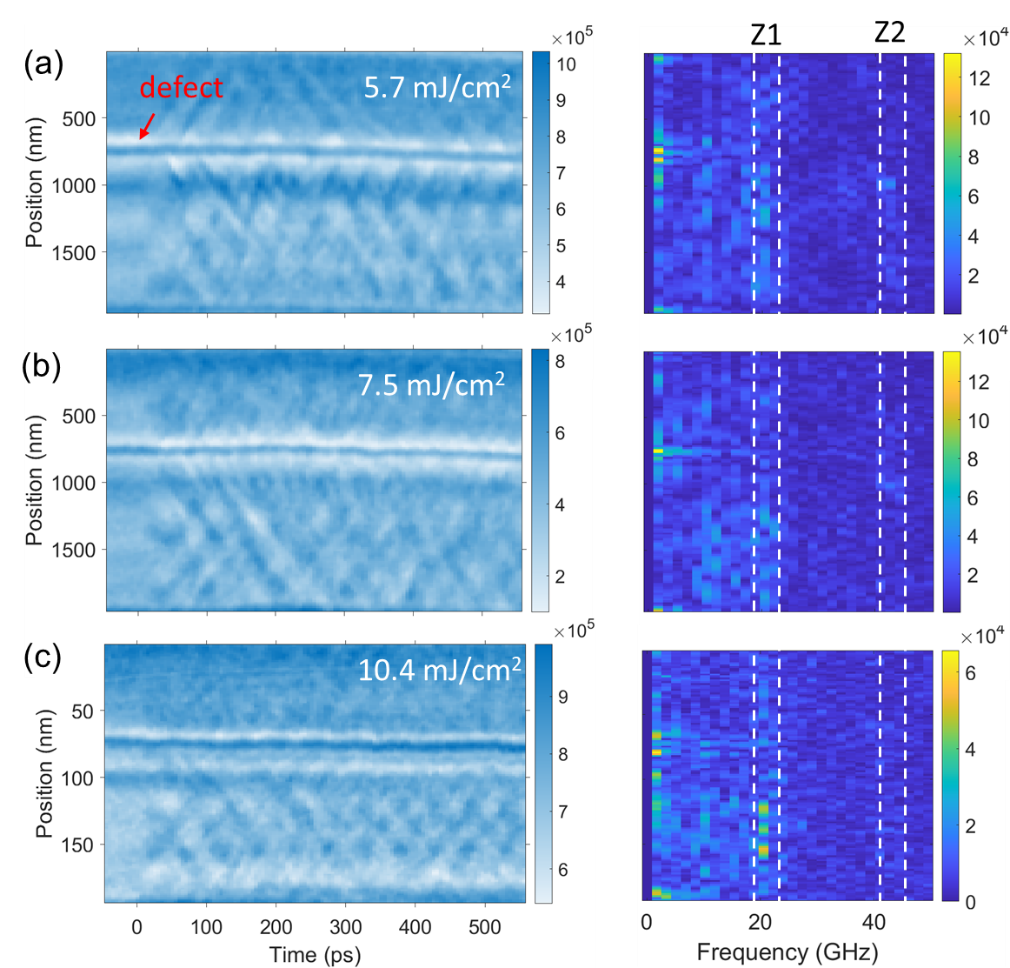


Figure S5. Fluence-dependent study of TOG driven phonon cavity. (a)-(c) are STCP from 27 nm thickness PdSe_2_ excited by 545 nm periodicity TOG with different average laser fluence as indicated in figure, with corresponding frequency map. Fundamental and second harmonic z mode are highlighted with dashed rectangles. The similar phonon signal suggests that the extracted phonon signal is independent from the thermal effect.

**Selected area electron diffraction measurement**


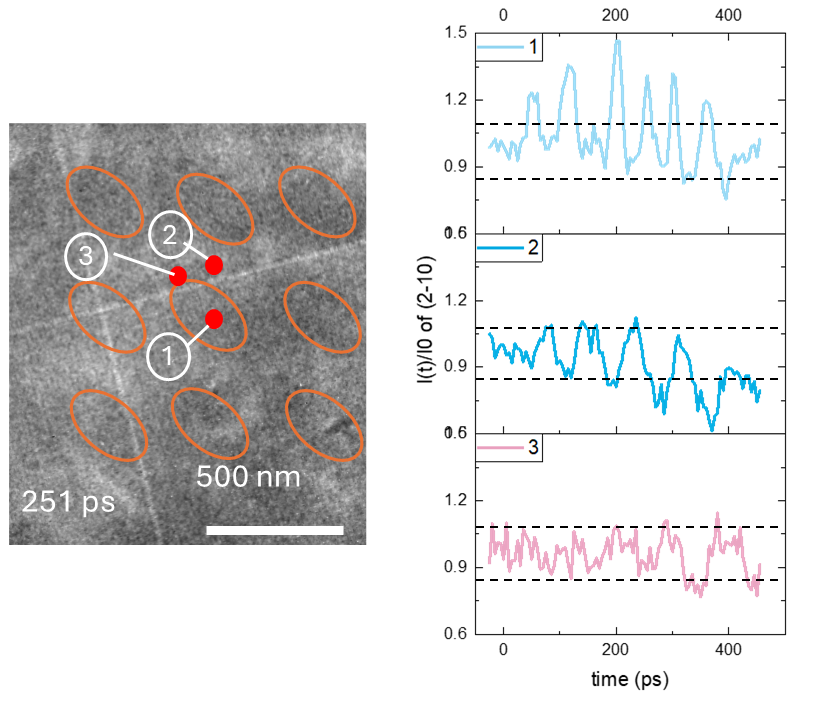


Figure S6. Selected area electron diffraction measurement. Left: the sample image with areas selected for diffraction highlighted with dots and numbered. The selected area for diffraction is a circular region with a diameter of 250 nm. Right: diffraction intensity temporal plot. Region 3 between hot and cold regions is quasi static. Regions 1 and 2 correspond to the center and edge of a phonon cavity, respectively.

**Simulation method and results**


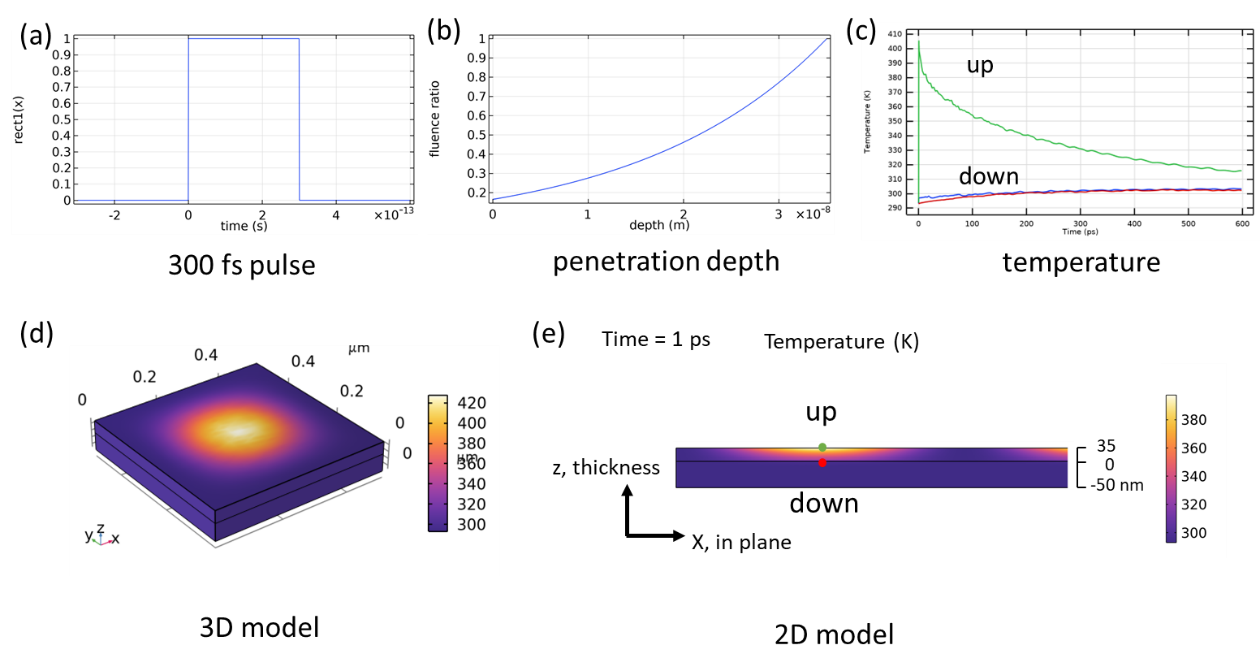


Figure S7. (a) the temporal profile of heat source used in the simulation. (b) the heat source profile in z direction. The normalized power decreases from top surface (z = 35 nm) to the bottom at z = 0, follow Beer–Lambert law. (c) temperature temporal profile extracted at positions (green and red solid circles) indicated in (e) at 1 ps following heat source applied. (d, e) 3D (d) and 2D (e) model constructed in the simulation, plotted with the temperature distribution at 1 ps time delay.

COMSOL Multiphysics is employed to simulate the structural dynamics of a PdSe_2_ sheet with different thickness with structured laser excitation. The Solid Mechanics and Heat transfer in solid modules were utilized for this study. The parameters for the material PdSe_2_ used in the simulation can be found in Table S2. The laser induced lattice heating is simulated by a transient heat source, which represents the energy transferred, per unit volume, from the laser pump to the material (a heat generation). The temporal profile of the heat source adheres to the temporal pulse length of the pump laser and is modelled as a 300 fs pulse applied from time 0 as shown in Figure S7(a). The local power of the heat source is modelled with a gradient in the thickness direction as shown in Figure S7(b), to simulate local light absorption modelled by Beer–Lambert law in the thickness direction. The power of the heat source follows a sinusoidal function along the x (x and y) axis for TOG (TOL) in the 2D (3D) model. The heat source leads to a lattice temperature rise of 100 °C as show in Figure S7(c).

3D and 2D model were constructed with a PdSe_2_ flake and a 50 nm Si_3_N_4_ substrate with the interface located at z = 0 nm as shown in Figure S7(e). The side boundaries are set in periodic conditions to simulate infinite sample size. The top and bottom boundaries are left free.

Table S2 material properties of PdSe_2_. Note the coefficient of thermal expansion is adopted from PtSe_2_.

| **Property** | **Value** | **Unit** | **Reference** |
| --- | --- | --- | --- |
| Density | \|  \| 6560 \| \| --- \| --- \| | kg/m³ | [2] |
| Thermal conductivity | k11=16,  k12=22,  k13=1.5 | W/(m·K) | [3] |
| Coefficient of thermal expansion | 31e-6 | 1/K | [4] |
| Heat capacity at constant pressure | 700 | J/(kg·K) | [5] |
| Absorption coefficient | 5.1579E7  (at 515 nm) | 1/m | [6] |


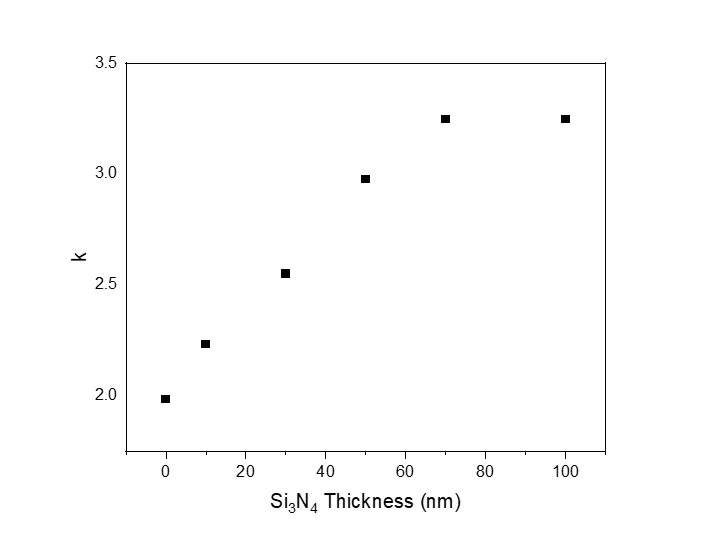


Figure S8 simulation of fundamental z mode with different substrate thickness. k is calculated by $\lambda=kL/n$, where L is the sample thickness, which is 35 nm in this case, n takes 1 for fundamental harmonics. wavelength of the wave is calculated by $\lambda=v/f$, where v is 1.56 nm/ps from Table S3, f is oscillation frequency from FFT spectrum.


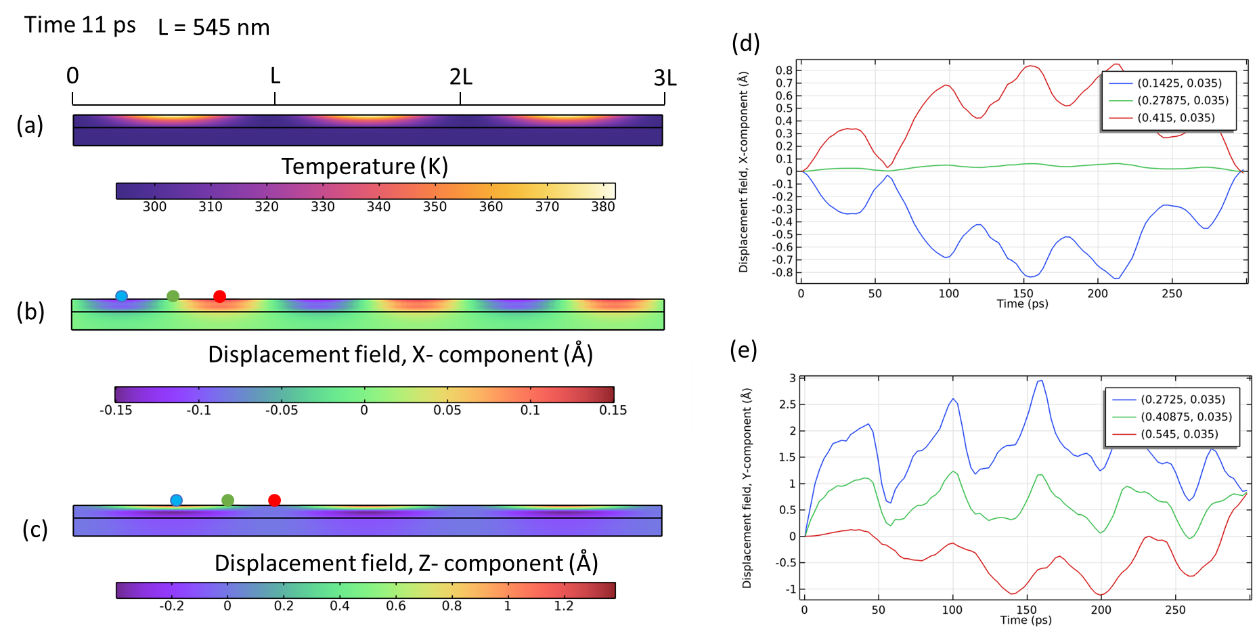


Figure S9. Simulated temperature (a) and displacement field in X (b) and Z (c) component in a 2D model at 11 ps time delay. Temporal trace of the displacement fields in X (d) and Z (e). The different traces are from positions indicated by solid circles with corresponding colors in (b) and (c). The plots show that the displacement field in X component is symmetric around maximum fluence position, while the displacement field in Z component gradually decreases from maximum to minimum position.


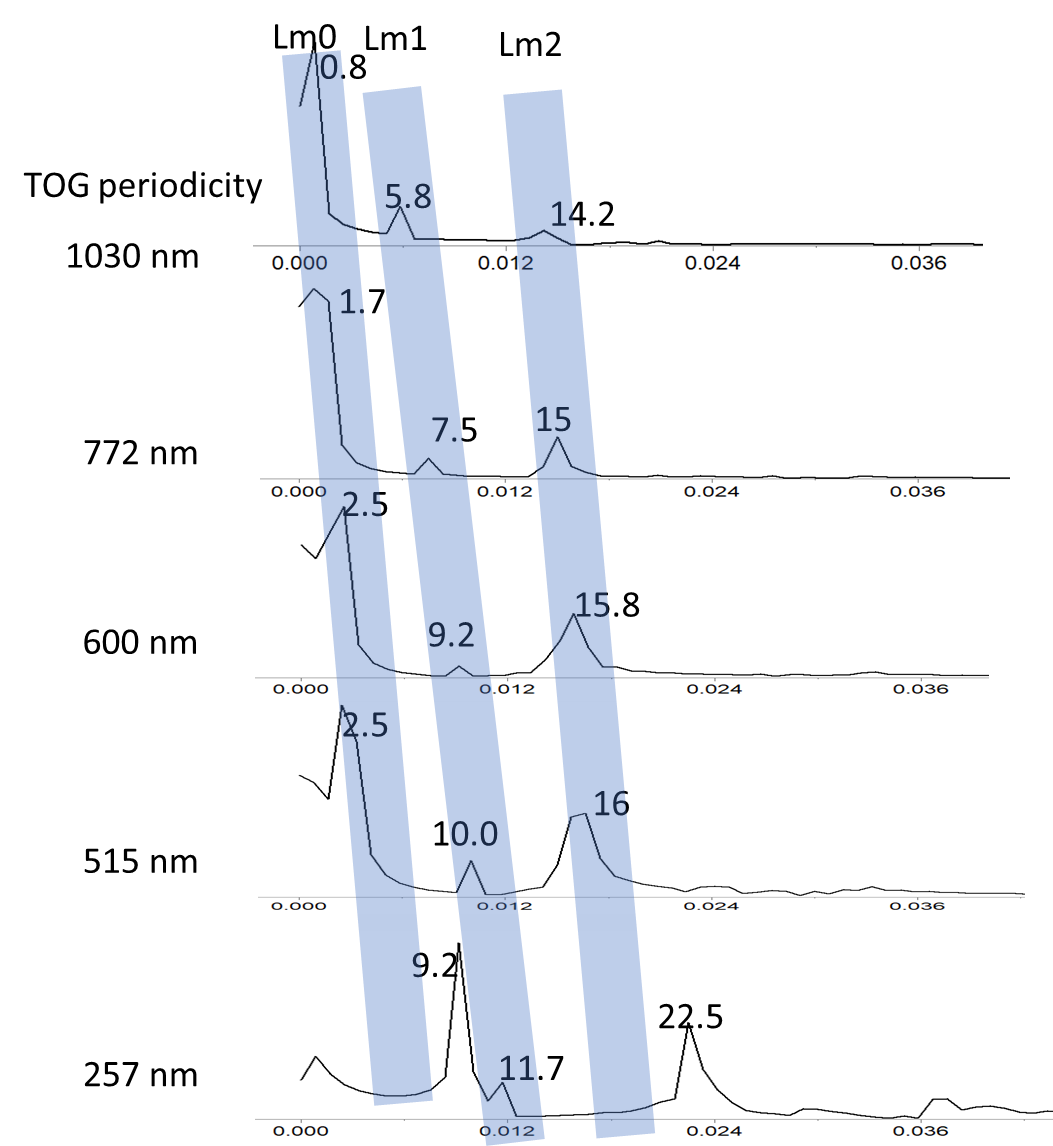


Figure S10 Frequency spectrum of structure oscillations from finite element simulation. The time-dependent X displacement component is extracted at the top surface with maximum fluence in the 2D model, and FFT is performed to get the frequency spectrum. The sample with 35 nm thickness is pumped by TOGs with different spatial periodicity as labeled at left. Three Lamb wave branches are highlighted with frequencies labeled with unit of GHz.


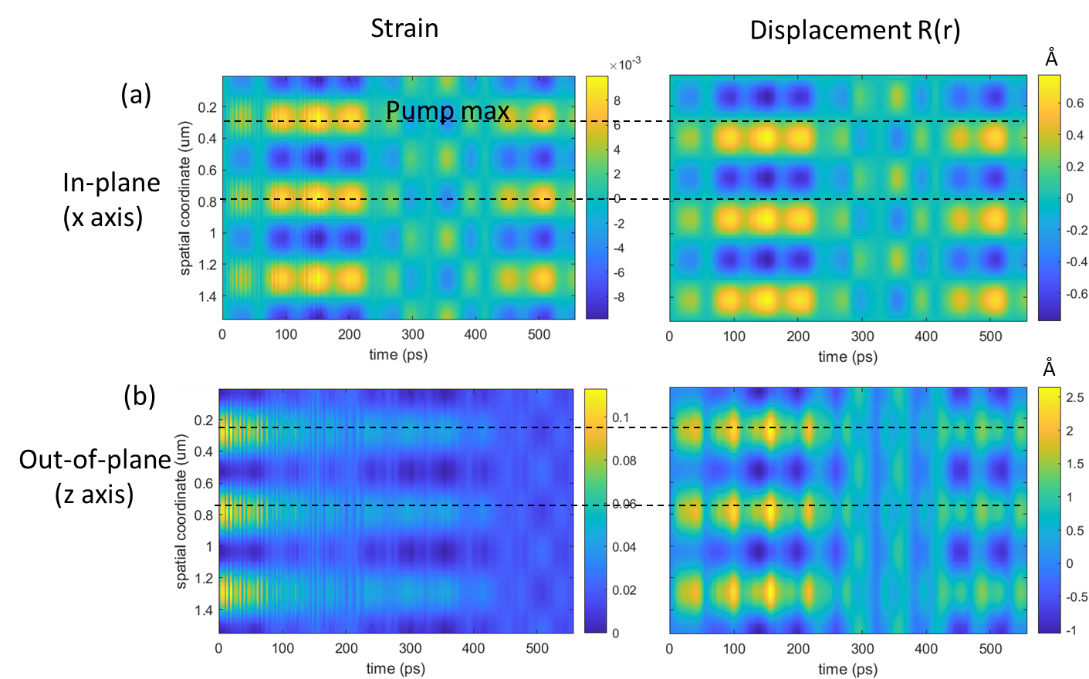


Figure S11. (a) Simulated strain (ε_xx_), in-plane displacement and (b) strain (ε_zz_), out-of-plane displacement. The data for stain and displacement is extracted along the top surface of the 2D model (Figure S7(e)) for each time delay. Dashed lines indicate the positions with maximum pump fluence, and a red box highlights a phonon cavity in space-time domain.


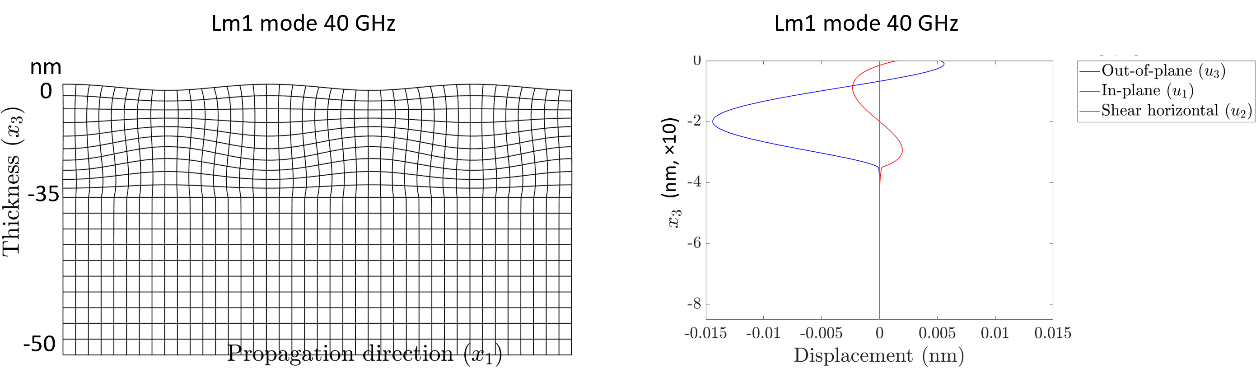


Figure S12. Displacement of 1^st^ Lamb mode. Left: figure illustration of displacement for a 35 nm PdSe_2_ sample on a 50 nm Si_3_N_4_ substrate. Right: line profiles of displacement in different direction. The data is calculated by Dispersion Calculator.[7]
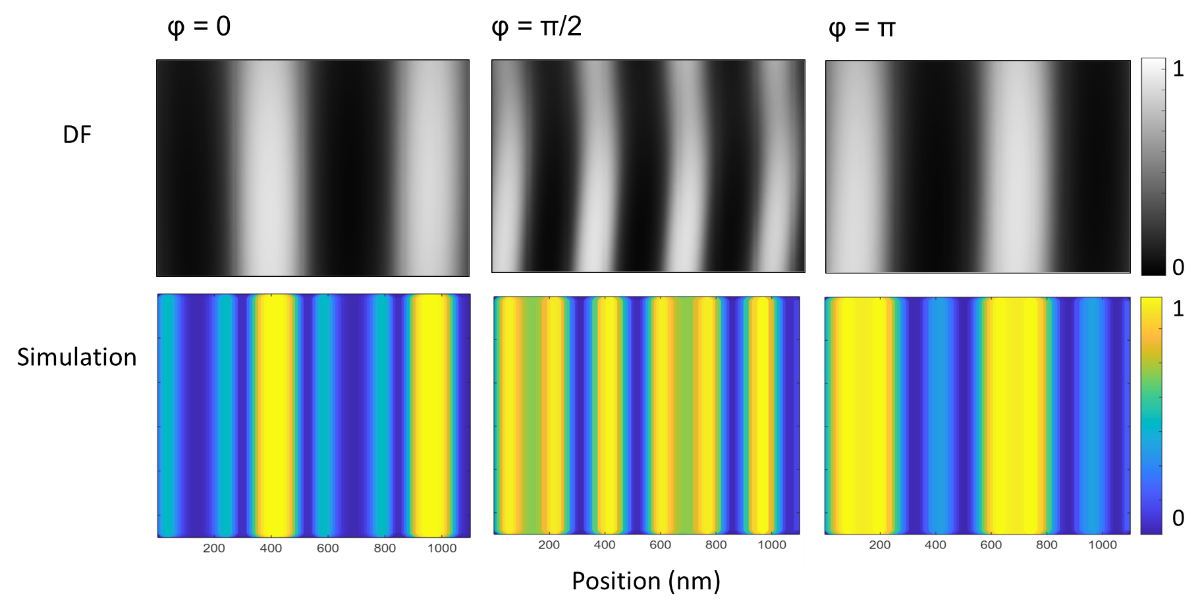


Figure 13. Simulated image contrast compared with experimental dark field images (FFT filtered). The additional side bands arise from the deviation of calculated excitation errors in static state from real experimental conditions.

Table S3 Calculated wave velocity of PdSe_2_ based on elastic constant from reference [2].


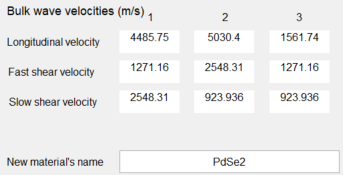


**Additional videos**

Movie S1. UEM dark field images, Bragg spot (210), from a flat PdSe_2_ sample under TOG excitation. The period of the TOG is approximately 545 nm. The PdSe_2_ flake is attached to a 50 nm thickness Si_3_N_4_ substrate. The projected TOG is parallel with the stripe contrast in the video.

Movie S2. UEM bright field imaging of a PdSe_2_ sample with different thicknesses under TOG excitation. The sample consists of three domains with thicknesses at 27 nm, 35 nm and 56 nm, respectively. The period of TOG is approximately 545 nm. The PdSe_2_ flake is attached on a 50 nm thick Si_3_N_4_ substrate.

**References**

1. Barot, J.B., S.K. Gupta, and P.N. Gajjar, *Optical properties of WTe2-a layered topological insulator: A DFT study.* Materials Today: Proceedings, 2023.

2. Zhang, K.C., et al., *Thickness-dependent anisotropic transport of phonons and charges in few-layered PdSe_2_.* Phys Chem Chem Phys, 2021. **23**(34): p. 18869-18884.

3. Xu, K., et al., *Unravelling the origin of thermal anisotropy in PdSe_2_.* 2D Materials, 2024. **11**(4).

4. Kjekshus, A., et al., *High Temperature X-Ray Study of the Thermal Expansion of PtS_2_, PtSe_2_, PtTe_2_ and PdTe_2_.* Acta Chemica Scandinavica, 1959. **13**: p. 1767-1774.

5. Li, M., et al., *Abnormal Thickness-Dependent Thermal Transport in Suspended 2D PdSe_2_.* Small, 2024: p. e2311125.

6. Polyanskiy, M.N., *Refractiveindex.info database of optical constants.* Scientific Data, 2024. **11**(1): p. 94.

7. Huber, A., *ArminHuber/Dispersion-Calculator: Dispersion Calculator v3.1*. 2025: Zenodo.
